# Supplementary material for: Contributions of gonadal hormones in the sex-specific organization of context fear learning
Source: PLoS One. 2023 Mar 2;18(3):e0282293. doi: 10.1371/journal.pone.0282293 (PMC9980802; doi:10.1371/journal.pone.0282293)
Supplement: S1 Fig — Motion index during the 2 secs preceding and during the initial footshock was compared in an analysis of covariance comparing sham male and female groups as well as sham-operated and gonadectomized sex-matched groups. No significant (ns) differences were identified (Sex: F 1, 35 = .09, p > .05; oRX: F 1, 15 = .142, p > .05; oVX: F 1, 17 = 3.168, p >.05). (DOCX) [file pone.0282293.s001.docx]

**Supplemental information**

**Supplemental Results:**

There is considerable variability in sham males between the separate experiments depicted in figure 2. It should be noted that in b and d, the male rats underwent conditioning on postnatal age (P) day 60, while in f, males underwent conditioning on P90. We have previously observed lower context fear conditioning in P90 than in P60 day-old adult male rats (Colón et al. 2018). However, given the design of the experiment, those data were never statistically compared. In running this analysis (univariate ANOVA), we did find a main effect of the Experiment between sham males depicted in figure 2 (F (2, 18) = 9.873, p = .001). However, these post-hoc comparisons (Bonferroni corrected) indicated that this main effect was driven by age differences in freezing (p < .001) between P90 (depicted in figure 2f) and P60 shams (figure 2d) and not among age-matched P60 shams (p > .05).

**Supplemental Figures**

**Figure S1 (caption):** Reactivity to footshock in adult rats did not differ between aggregate male and female groups nor between neonatal surgical and sham-operated groups. Motion index during the 2 secs preceding and during the initial footshock was compared in an analysis of covariance comparing sham male and female groups as well as sham-operated and gonadectomized sex-matched groups. No significant (ns) differences were identified (Sex: F _1, 35_ = .09, p > .05; oRX: F _1, 15_ = .142, p > .05; oVX: F _1, 17_ = 3.168, p >.05).

**Figure S1:**
