# Supplementary material for: Contributions of gonadal hormones in the sex-specific organization of context fear learning
Source: PLoS One. 2023 Mar 2;18(3):e0282293. doi: 10.1371/journal.pone.0282293 (PMC9980802; doi:10.1371/journal.pone.0282293)
Supplement: S1 File — (PPTX) [file pone.0282293.s002.pptx]

## Slide 1
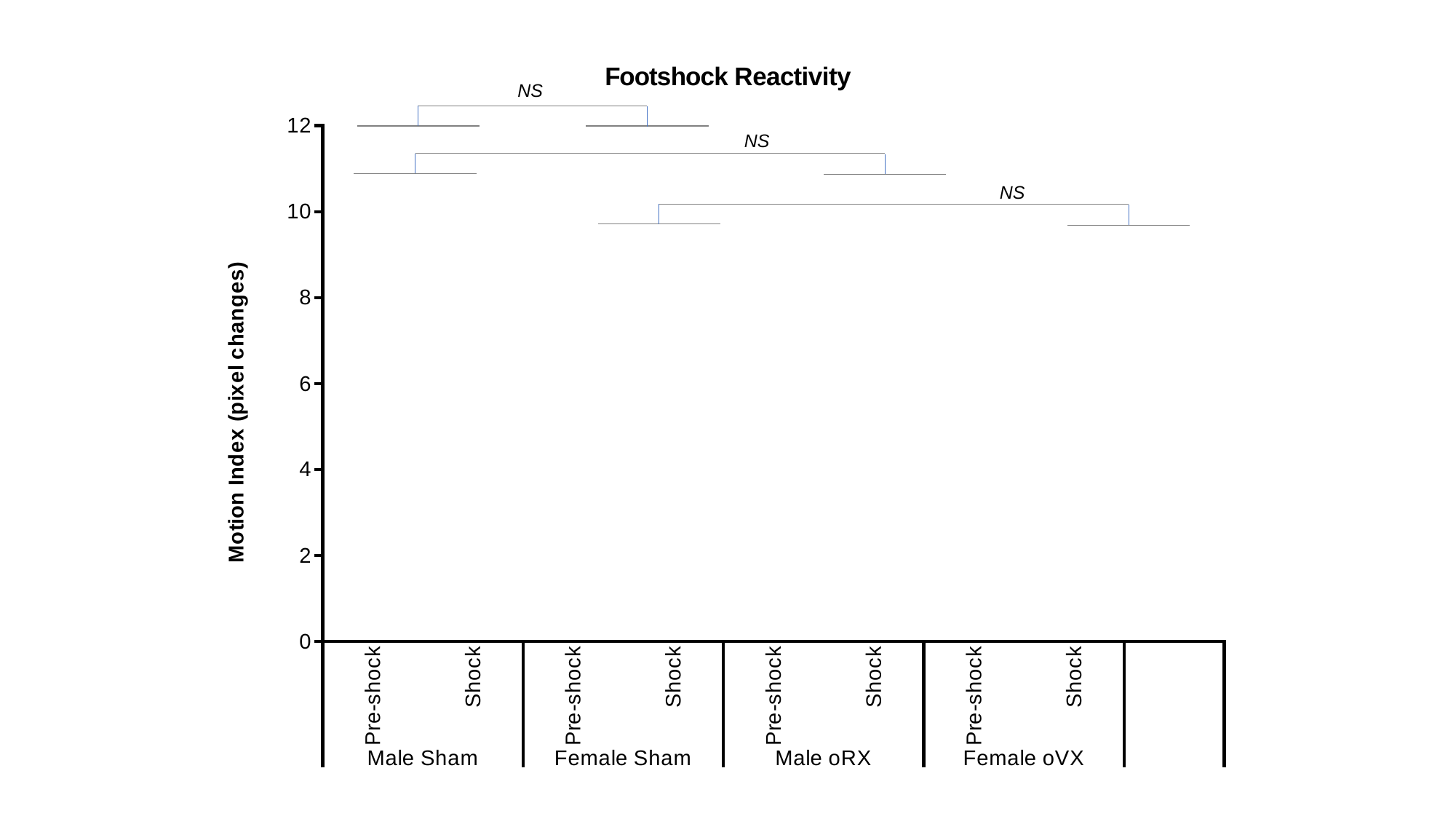

### Chart: Footshock Reactivity
| Category | Mean |
|---|---|
| Pre-shock | 1288.12 |
| Shock | 6534.06 |
| Pre-shock | 823.21625 |
| Shock | 5605.688749999999 |
| Pre-shock | 1310.5090000000002 |
| Shock | 6099.969 |
| Pre-shock | 1335.9985714285717 |
| Shock | 5171.160000000001 |NS
NS
NS
